# Sexual and reproductive health literacy of higher education students: a scoping review of determinants, screening tools, and effective interventions

**DOI:** 10.1080/16549716.2025.2480417

**Published:** 2025-03-21

**Authors:** Nour Waleed Zuhair Alhussaini, Usra Elshaikh, Khadiga Abdulrashid, Sana Elashie, Noor Ahmed Hamad, Ghadir Fakhri Al-Jayyousi

**Affiliations:** a Department of Public Health, College of Health Sciences, Qatar University, Doha, Qatar; b World Innovation Summit for Health, Qatar Foundation, Doha, Qatar; c Department of Health Education, Primary Healthcare Corporation, Doha, Qatar

**Keywords:** Sexual health, reproductive health, health literacy, higher education, screening tools, culture, religion, technology-based interventions, scoping review

## Abstract

Health literacy is a determinant of overall health, particularly among college students who are at increased risk for negative sexual and reproductive health outcomes. Contextualized sexual and reproductive health education serves as an important protective measure for this population. This scoping review examines sexual and reproductive health literacy among college students to identify key determinants, screening tools, and effective interventions. Following the PRISMA-ScR guidelines, a comprehensive systematic search was conducted through PubMed, Embase, and Scopus databases without restrictions to capture all publications related to health literacy of students of higher education from January 2013 to March 2023. Out of 4,526 articles identified in the initial search, 11 studies met the inclusion criteria for this review. Most studies were cross-sectional and conducted in the USA. Sexual and reproductive health literacy was examined as both an outcome, influenced by factors like age, gender, religion, study area, sexual education, birthplace/region, and race/ethnicity, and as an exposure shaping knowledge, attitudes, and behaviors regarding sexual and reproductive health. On the interpersonal level, family influence played a crucial role in shaping sexual and reproductive health literacy. The review also found correlations between health literacy and knowledge, attitudes, and practices, with technology-based interventions. Based on the findings, a conceptual model was developed. Research on sexual and reproductive health literacy remains limited, particularly in sensitive sociocultural contexts. Further studies are needed to explore the impact of sociocultural, religious, and environmental factors on young people’s health literacy in order to inform more effective interventions.

## PAPER CONTEXT

- **Main findings**: Sexual and reproductive health literacy influences knowledge, attitudes, and behaviours related to sexual and reproductive health. Technology-based interventions show potential to improve health literacy and related outcomes.
- **Added knowledge**: Given the scarcity of research on sexual and reproductive health literacy among college students from diverse sociocultural contexts, this scoping review underscores the potential role of family, and also technology-driven solutions, in enhancing health literacy and informing effective interventions.
- **Global health impact for policy and action**: The findings call for culturally tailored education programs and emphasize the critical role of family and community dynamics in sexual and reproductive health literacy interventions. Strengthening these aspects will help promote global health equity in this area.

## Background

Sexual health is defined as ‘a state of physical, emotional, mental, and social well-being in relation to sexuality; it is not merely the absence of disease, dysfunction, or infirmity’ [1]. It is a vital component of both physical and mental wellness. Healthy relationships and sexual satisfaction are essential aspects of good sexual health, along with access to information and sexual health services [2]. Sexual and reproductive health (SRH) encompasses the ability to prevent unplanned pregnancies, unsafe abortions, and sexually transmitted infections (STIs), as well as to avoid all forms of sexual assault, violence, and coercion [3].

Health literacy (HL) is widely recognized as a key social determinant of health and a crucial factor in the success of health education initiatives [4]. HL is defined as ‘the degree to which individuals have the ability to find, understand, and use information and services to inform health-related decisions and actions for themselves and others’ [5]. Additionally, it includes critical and interactive skills necessary for individuals to manage their health effectively. HL is considered a public health asset that can reduce health inequities and enhance social capital [4]. By integrating education and health, HL provides a framework that strengthens people’s ability to maintain good health throughout their lives [4]. Low health literacy is associated with poorer healthcare utilization, worse health outcomes, higher healthcare costs, and greater disparities in health [6].

Building on the concept of health literacy, sexual and reproductive health literacy (SRHL) refers to an individual’s self-perceived capacity to obtain, comprehend, evaluate, and apply information to make informed decisions that positively impact SRH. SRHL extends beyond knowledge and behavior to include the thought process behind SRH-related decisions [7].

Adolescence is a critical, rapidly evolving developmental stage marked by significant mental, physical, and behavioral changes, often accompanied by risk-taking behaviors [3]. It is a pivotal period for developing independent decision-making skills, making it essential to provide individuals with accurate and reliable health information to foster lifelong healthy behaviors [4]. College students, encompassing teenagers, adolescents, and young adults, are at higher risk of adverse SRH outcomes, including teenage pregnancy and STIs [8]. For instance, individuals aged 18–19 account for two-thirds of all teen pregnancies. Additionally, only about 40% of sexually active individuals in this age group report consistent and correct contraceptive use [8].

Teenage pregnancy remains a prevalent issue, linked to adverse maternal and infant outcomes. It is associated with increased risks of unsafe abortion, gender-based violence, and maternal mortality among young mothers [9]. Moreover, teenage pregnancy has long-term consequences, such as a decreased likelihood of completing education and diminished economic well-being [10]. According to the Centers for Disease Control and Prevention (CDC), STI rates are highest among college-aged individuals, with approximately 50% of all new STIs occurring in the 15–24 age group each year [8]. STIs can result in infertility, chronic pelvic pain, and an increased risk of HIV transmission by up to eight times [11,12]. For instance, 10–15% of sexually active individuals with chlamydia may develop pelvic inflammatory disease (PID) if untreated, potentially leading to infertility due to irreversible reproductive organ damage [13]. Low SRHL is believed to contribute to these adverse health outcomes by influencing poor SRH-related decisions.

Among young adults, the Internet is a primary source of sexual health information. Web-based sexual health interventions may be more effective than traditional in-person interventions, as they can reach a broader audience at a lower cost while ensuring privacy and confidentiality [14]. A 2017 study reported that verbal education combined with active participant involvement in discussions was an effective strategy for promoting SRHL [15]. Another study recommended comprehensive interventions focusing on stigma reduction, positive messaging, and the development of critical and interactive skills to enhance SRHL. It also emphasized the importance of contextual and key factors, such as access to official health services and accurate online information [16]. Thus, the organization and accessibility of appropriate and relevant sexual health services play a crucial role in the provision and promotion of SRHL [16].

Health literacy is context-dependent, meaning different settings may require different assessment tools. The Rapid Estimate of Adult Literacy in Medicine (REALM) and the Test of Functional Health Literacy in Adults (TOFHLA) are commonly used HL assessment tools originally developed for clinical settings [17]. However, these tools have been criticized for their limited scope, as they primarily assess reading and numeracy skills while neglecting other critical aspects of HL, such as critical thinking, communication, and self-efficacy [17]. Nonetheless, several specific instruments have been designed to assess young adults’ HL. This study aims to summarize the most common tools and interventions used to assess and improve SRHL among young adults.

No comprehensive reviews have been conducted on SRHL among higher education students. Therefore, the purpose of this review is to map the available literature on SRHL in university students and identify key determinants, screening tools, and interventions. This review will explore how HL functions as both an outcome and a determinant in SRH, highlight gaps in the literature, recommend appropriate screening tools to assess university students’ SRHL needs, and identify effective interventions for implementation in university settings.

## Methods

### Study design

This study employs a scoping review methodology, following the Preferred Reporting Items for Systematic Reviews and Meta-Analyses extension for Scoping Reviews (PRISMA-ScR) guidelines [18]. It is part of a larger project comprising multiple reviews on health literacy.

### Search strategy

A comprehensive systematic search was conducted in the PubMed, Embase, and Scopus databases, covering publications from January 2013 to March 2023. The search strategy included two primary terms: ‘Health Literacy’ AND ‘University student*,’ combined using the AND Boolean operator. The ‘university student’ term encompassed multiple keywords, as presented in Table 1, and these were connected using the OR Boolean operator. The search in all databases was restricted to studies published within the last 10 years. The full search strategy for each database is detailed in Supplementary Table S1.

**Table 1.** Search strategy.

Search #	Search terms
#1	Health Literacy
#2	College OR University OR Universit* OR “University student*” OR “Undergraduate student*” OR “Postgraduate student*” OR “College student*” OR “Tertiary student*” OR “Higher education” OR “Tertiary education”
#3	#1 and #2

### Study selection

All identified studies were imported into EndNote software for duplicate identification and removal. Remaining articles were uploaded to Rayyan where additional duplicates were manually removed. When encountering duplicates, the most updated articles containing information relevant to our study were retained.

This study employed a two-level title/abstract screening process. In the first level, following identification of studies for screening, articles were distributed among six public health students and the corresponding author. Throughout the study selection and assessment process, the corresponding author was responsible for resolving disagreements and conducting final evaluations. The following criteria were used to assess study eligibility for the first level of screening:

### Inclusion criteria

- Explicit mention of health literacy concept
- Population consisting of higher education students
- Publications in English
- Primary research studies

### Exclusion criteria

- Reviews, conference abstracts, theses, dissertations, gray literature, and news outlets

Thematic labeling was conducted by five authors (NWZA, UE, NH, SA, GFA) along with public health students, categorizing articles according to the health topics they addressed in relation to health literacy. The finalized major health literacy topics included sexual and reproductive health literacy (SRHL), general health literacy, mental health literacy, behavior and lifestyle health literacy, and digital health literacy. Articles irrelevant to these identified main thematic labels were excluded from this project.

**Figure 1.**
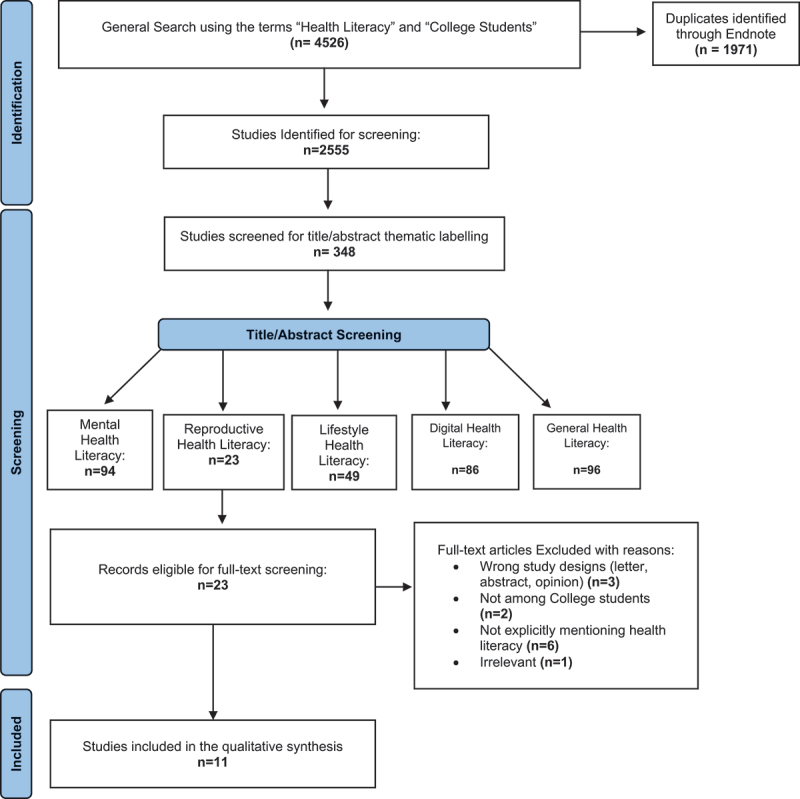
PRISMA flowchart.

In the second level of title/abstract screening, articles labeled under the SRHL theme were considered for this scoping review. The first and second authors screened the abstracts of these articles and excluded studies that did not explicitly mention health literacy, did not include higher education student populations, or were not primary research studies. Reference lists of eligible articles were also searched to ensure inclusion of all relevant studies. Figure 1 displays the PRISMA flowchart of included studies.

#### Data extraction

A spreadsheet was created prior to beginning data extraction. The spreadsheet included the following information to be extracted from each article: first author, year, title, study design, aim/objectives, country, type of reproductive health topic, age in years, gender percentage, sample size, SRHL intervention (if applicable), SRHL measurement tool used, other tools used, SRHL correlation with other variables, SRHL as outcome, main determinants (when SRHL was outcome), significantly associated determinants of SRHL, SRHL as exposure, outcome (when SRHL was exposure), SRHL predicting outcome (significantly associated), and main findings. After initial creation, the data extraction spreadsheet was pilot tested by all authors to ensure accuracy and relevance, and underwent adjustments and refinements as needed throughout the pilot process. All five authors extracted data from included studies, and the first author verified the extracted data to ensure accuracy of findings.

#### Data synthesis

Data were summarized in tables and figures, with descriptive data presented as frequencies, percentages, or ranges as appropriate. A conceptual model was also created to represent the main findings of this review.

## Results

### Characteristics of included studies

Among the 11 included studies, the majority (8 studies) employed a cross-sectional approach [19–26]. The remaining studies included two qualitative investigations [27,28] and one randomized controlled trial [29]. The combined sample size across all studies totaled 7,936 participants. Geographically, studies were predominantly conducted in the USA (six studies) [19–21,24,27]. Other locations included Australia (two studies) [22,23], and one study each in Japan [25], Thailand [26], and China [29]. Notably, no research was reported from Europe, South America, Africa, or the Middle East and North Africa regions.

The studies covered a diverse range of topics: four focused on Human Papillomavirus (HPV) [19–21,25]; one examined basic reproductive biology, sexually transmitted infections (STIs), and unplanned pregnancy [22]; and another investigated comprehensive sexual health knowledge including HIV/Hepatitis, STIs, pregnancy, female reproduction, contraception, and STI prevention [23]. Additional studies explored the prevention of unintended pregnancies, overall sexual and reproductive health (SRH), contraception use and STI prevention, and condom use [24,26–29]. Participants across studies ranged in age from 17 to 35 years. Females were predominantly represented, forming the majority in seven studies [19,20,22–24,27,28], serving as the exclusive focus in three studies [21,26,28], and constituting less than 50% in one study [25]. Health literacy was assessed in nine articles as exposure, outcome, both, or was correlated with other variables (Table 2). Table 3 presents a summary of characteristics of included studies.

**Table 2.** Health literacy as exposure, outcome, or correlation with other variables in included articles.

First author, year of publication	Correlation with other variables	RHL as exposure	RHL as outcome
Amy E Albright, 2018	x	x	x
Harriet Kitur, 2022	x		
Hee Yun Lee, 2015		x	
Steve Simpson Jr, 2017			x
Steve Simpson Jr, 2015			x
Ashley Sons, 2023	x		x
Tomoya Suzuki, 2022		x	
Saowanee Thongnopakun, 2018		x	x
Janet Yuen-Ha Wong, 2021		x

**Table 3.** Characteristics of included studies.

First author	Year	Study design	Aim of study	Country	Topic	Age (years)	Female %	SS
Ashley Sons	2023	CS	To examine the correlation between health literacy and basic knowledge of reproduction, contraception, and STIs in female college students	USA	Female reproduction, contraception, and STI	18–24	73.4%	323
Harriet Kitur	2022	CS	To assess university students’ knowledge and understanding about HPV and its association with vaccination status and health literacy	USA	HPV	18–26	66.8%	383
Tomoya Suzuki	2022	CS	To identify the effects of knowledge, health literacy, and health belief-related factors on vaccine intention among university students	Japan	HPV	18–35	46%	318
Cheryl A. Vamos	2022	Qual	To explore health literacy needs and preferences for a technology-based intervention (app) to improve SRH among college students	USA	SRHL	≥18	50%	20
Janet Yuen-Ha Wong	2021	RCT	To describe the web-based sexual health literacy intervention called “Smart Girlfriend” for female university students	China	Condom use	18–32	100%	781
Cheryl A Vamos	2020	Qual	To assess self-perceived SRHL (contraception use and STI prevention) among college students	USA	Contraception use and STI prevention	≥18	70%	43
Amy E Albright	2018	CS	To assess HPV health literacy in college students	USA	HPV	≥18	58.1%	360
Saowanee Thongnopakun	2018	CS	To determine whether there is an associated effect between sociodemographics and sexual risk behaviors with health literacy in preventing unintended pregnancy among university students	Thailand	Prevention of unintended pregnancy	18–24	100%	418
Steve Simpson Jr	2017	CS	To determine the relationship of attitudinal factors with sexual health literacy score; and the impact of adjusting for attitudinal factors on the relationship of demographic and behavioral covariates with sexual health literacy	Australia	Basic reproductive biology and STI and unplanned pregnancy	≥17	65.1%	1234
Hee Yun Lee	2015	CS	To investigate predictors of HPV vaccination in young Asian American and Pacific Islander and non-Latina white women	USA	HPV	18–25	100%	2270
Steve Simpson Jr	2015	CS	To evaluate sexual health literacy among students at university of Tasmania	Australia	Knowledge on sexual health, HIV/Hepatitis, STI, and pregnancy	≥17	62.8%	1786

SS= sample size, CS= Cross-sectional study, Qual= Qualitative study, RCT= Randomized controlled trial, STI= sexually transmissible infections, SRH= Sexual and reproductive health, SRHL= Sexual and reproductive health literacy.

### Health literacy as an outcome

Six studies included health literacy as an outcome. Of these, three studies assessed health literacy related to reproductive/sexual health topics (sexual diseases, HIV/hepatitis, HPV), basic reproductive biology, contraceptives, STIs, or unplanned pregnancies. One study exclusively assessed health literacy outcomes regarding HPV, and another focused solely on unintended pregnancy. Three articles examined demographics as predictors of health literacy, while all five quantitative studies investigated specific sexual health-related behaviors as predictors of health literacy. One qualitative study assessed college students’ SRHL, emphasizing STI prevention and contraception. Key findings revealed that while visual aids and trustworthy individuals enhanced knowledge, the Internet remained a primary but occasionally inaccurate information source [28].

Results from the included studies showed that the majority of participants (95%) demonstrated adequate literacy, while a small percentage had possible limited literacy or high likelihood of limited literacy (5%) [24]. One study indicated that most students possessed adequate sexual health literacy [23], and another study found that 80% had adequate health literacy [19]. When assessing health literacy through confidence in form completion, approximately 42% reported being ‘extremely confident’ or ‘quite a bit confident,’ while 58% indicated they were ‘sometimes confident,’ ‘a little confident,’ or ‘not at all confident’ [20]. In the study conducted by Thongnopakun and colleagues, approximately 52% reported appropriate health literacy scores for preventing unintended pregnancy, while 48% had inappropriate health literacy scores [20].

Certain studies identified factors contributing to higher health literacy among college students, including female sex, age above 20 years, sexual orientation, certain religions (e.g. Catholic), field of study (Medicine, Nursing, History, Languages, and Allied Health), age of first sexual experience, year in school, sex education, number of sexual partners, and sexual experience. Higher SRHL was observed among students pursuing degrees in allied health, nursing, and medicine – fields where sexual health is frequently discussed and sex education is promoted.

Conversely, some studies highlighted factors that negatively affected SRHL among college students. These factors primarily related to participants’ region, race/ethnicity, birthplace, and religion. Regionally, students from China, Malaysia, India, Pakistan, Africa, and other East and South Asian nations demonstrated lower SRHL. Students who identified as Protestant, Muslim, Hindu, or Buddhist also had lower SRHL, suggesting that religious beliefs impact literacy levels. These religious communities may hold more conservative values related to sexuality and reproductive health, potentially restricting open communication and instruction.

Moreover, family discussions related to sexual health were negatively associated with SRHL, as these topics were often considered taboo. Table 4 presents significant determinants of health literacy before and after adjusting for confounders.

**Table 4.** Sexual and reproductive health literacy as an outcome.

First author	Year	Health literacy measurement tool	Determinants of health literacy
Ashley Sons	2023	NVS**	Positive association with sexual health literacy scores: never had previous pregnancies and cisgender (gender identity aligns with sex assigned at birth)†
Cheryl A Vamos	2020	N/A	Key findings included medical language as a barrier for SRHL, graphics aiding comprehension, and the Internet as the primary information source. Symptoms, family and friend guidance, and lifestyle were all included in the appraisal. Through seeking medical attention and interacting with peers and professionals, participants put their knowledge into practice.
Amy E Albright	2018	HLQ* and NVS**	Positive association with NVS score: Male sex, Familiarity with HPV, and its vaccines (compared to familiarity with vaccine only group, and non-familiarity with HPV and vaccines group), and familiarity with HPV only (compared to familiarity with vaccine only group, and non-familiarity with HPV and vaccines group) † Positive association with HLQ score: Familiarity with HPV and its vaccines (compared to familiarity with HPV only) in scale 1: Feeling understood and supported by healthcare providers†, Familiarity with HPV, and its vaccines (compared to familiarity with HPV only group, and non-familiarity with HPV and vaccines group) in scale 5: Appraisal of health information†
Saowanee Thongnopakun	2018	Health literacy scale for unwanted-pregnancy prevention created by the Ministry of Public Health	Positive association with health-literacy-promoting behavior to prevent unintended pregnancy: Current residence type (Dormitory in the university or staying with parents), no previous sexual intercourse, cognitive health to prevent unintended pregnancy, decision-making skills in choosing appropriate practice to prevent unintended pregnancy, and self-management to prevent unintended pregnancy ‡
Steve Simpson Jr	2017	Sexual health section in ARCSHS***Secondary Students and Sexual Health Survey (ARC/LaTrobe), and the University of Missouri Sexual Health Survey (SHS/Missouri)****	Positive association with sexual health literacy: Female sex^a,b^, Age >20^a,b^, homosexual orientation^a^, catholic religion^a^, study area (Allied health ^a,b^, medicine ^a,b^, nursing ^a^), age of first sex 10–17 years ^a^ and 10–40 years^b^ ‡ Negative association with sexual health literacy: Asia region^a,b^; east and south Asian race^b^; None/unaffiliated religion^b^, being Christian^b^ and buddhist^a,b^; and taboo subject in family sexual discussion^a,b^ ‡
Steve Simpson Jr	2015	Sexual health section in ARCSHS***Secondary Students and Sexual Health Survey (ARC/LaTrobe), and the University of Missouri Sexual Health Survey (SHS/Missouri)****	Positive association with sexual health literacy scores: Female sex; being >20 years old; homosexual orientation^a;^ study area (medicine^a,b^, nursing^a^, History & Languages^b^, and allied health^a,b^); 3rd school year^a,b^ or higher^a^, 5th year or higher^b^ and postgraduate years^b^; sexual education (at secondary school, university, and multiple)^a,b^; basic only^b^, basic and advanced^a,b^/+extra^a,b^ sexual education; only physical^a,b^ and both virtual and physical sexual experience ^a,b^; ever had sexual experience^a,b^; younger age at first sexual experience (10–17 years) ^a^ and any age of first sexual experience^b^; 0->50^a^ and 1 or more^b^ life number of sexual partners of opposite sex; 0–15, 21->50^a^, and 0–10^b^ Life number of sexual partners of same sex; Female sexual behavior: (Basic sexual behavior^b^, Basic sex toy use ^a,b^, homosexual behavior ^a,b^, Other sexual behavior ^a,b^), and Male sexual behavior: (Physical sex no detail ^a^, Basic sexual behavior ^a,b^, Basic sex toy use ^a,b^, Anal and homosexual behavior ^a,b^, Other sexual behavior ^a,b^)‡ Negative association with sexual health literacy scores: Questioning/Not sure sexual orientation^a^, Africans ^a,b^, south South Asians ^a,b^,and east East Asians ^a^; Unaffiliated but spiritual ^b^, Christian ^b^, Protestant ^a^, Islam ^a,b^, Hindu^b^ and Buddhist ^a,b^ religions; birthplace in Malaysia ^a,b^, india ^a,b^, Pakistan ^b^, China ^b^ ‡; sexual topic discussed as totally taboo subject in family ^a,b^ ‡

*44-item Health Literacy Questionnaire, ** Newest Vital Sign, *** Australian Research Centre in Sex, Health, and Society (ARCSHS), ****Sexual health knowledge component, †significant result but not adjusted, ‡ Significant result and adjusted for confounders, a= Associated with ACR scale score, b= Associated with NVS scale score.

### Health literacy as an exposure

Table 5 summarizes findings from selected studies addressing SRHL as a determinant/exposure shaping various SRH-related outcomes and the measurement tools applied in these studies. Using the Health Literacy Questionnaire (HLQ) and Newest Vital Sign (NVS), Albright and Allen [19] found that sexual health literacy was associated with HPV knowledge and confidence, HPV vaccine knowledge, and sexual attitudes. Another study by Hee Yun Lee applied a 5-item questionnaire developed and adapted from vaccination guidelines created by the National Cancer Institute. This study found that sexual health literacy was associated with HPV vaccination completion, and that HPV vaccine literacy was the only significant enabling predictor for the Asian American and Pacific Islander group [21].

**Table 5.** Sexual and reproductive health literacy as an exposure.

First author	Year	Health literacy measurement tool	Outcome	Health literacy is significantly associated with:
Tomoya Suzuki	2022	Self-developed questionnaire based on Ishikawa et al. study	Intention to HPV vaccination	Intention to HPV vaccination among unvaccinated males and females‡
Janet Yuen-Ha Wong	2021	NA***	Self-reported consistency of condom use. The secondary outcome was knowledge, attitudes, norms, and self-efficacy of condom use	The web-based sexual health literacy program did not significantly increase the consistency of condom use compared to a single webpage of condom use information†. It temporarily improved knowledge, attitudes, norms, and self-efficacy of condom use†
Amy E Albright	2018	HLQ* and NVS**	HPV knowledge, HPV vaccine knowledge, and sexual attitudes	Scale 1: Feeling understood and supported by healthcare providers of HLQ predictor of HPV knowledge† NVS score and HLQ scale 4: Social support for health predictor of HPV vaccine knowledge score† NVS associated with participant sexual attitudes: higher view of birth control is important component of sexuality†
Saowanee Thongnopakun	2018	Health literacy scale for unwanted-pregnancy prevention created by the MoPH	Unintended-pregnancy-preventive behaviors	Media and information literacy was significantly associated with unintended-pregnancy-preventive behaviors among university students†
Hee Yun Lee	2015	5-item self-developed questionnaire adopted from vaccination guidelines created by the NCI	HPV vaccination completion	HPV vaccine literacy was significantly associated with completion of HPV vaccine for NLW group‡ HPV vaccine literacy was the only significant enabling predictor for the Asian American and Pacific Islander group‡

*44-item Health Literacy Questionnaire, **Newest Vital Sign, *** Not applicable,NCI= National Cancer Institute MoPH= Ministry of Public Health.

†significant result but not adjusted, ‡ Significant result and adjusted for confounders.

Tomoya Suzuki developed a questionnaire based on Ishikawa et al.‘s study to examine the role of health literacy in influencing decisions regarding HPV vaccination. Results showed that health literacy was associated with the intention to get HPV vaccination among unvaccinated males and females [25]. Moreover, another study found that health literacy played a role in influencing unintended-pregnancy-preventive behaviors, with media and information literacy being significantly associated with these behaviors among university students. The health literacy scale for unwanted-pregnancy prevention created by the Ministry of Public Health was applied in this research study [26]. Janet Yuen-Ha Wong found that sexual health literacy predicted the reported consistency of condom use, knowledge, attitudes, norms, and self-efficacy of condom use. The web-based sexual health literacy program improved knowledge, attitudes, norms, and self-efficacy of condom use among study participants [29].

### Health literacy measurement tools

Eight studies in our review used different reproductive/sexual health literacy measurement tools [19–25]. In total, nine tools were used for quantitatively assessing reproductive/sexual health literacy, including the Health Literacy Questionnaire (HLQ), Newest Vital Sign (NVS), Sexual Health Knowledge Component of the Australian Research Centre in Sex, Health and Society (ARCSHS) Secondary Students and Sexual Health Survey (ARC/Latrobe), Sexual Health Knowledge component of the University of Missouri Sexual Health Survey (SHS/Missouri), Health Literacy Scale for Unwanted-Pregnancy Prevention developed by the Thailand Ministry of Public Health, self-developed questionnaires, and assessment of health literacy using one item (confidence level) when filling out medical forms. These measurements broadened and supplemented our understanding of university students’ SRHL.

In Albright and Allen’s study [19], HLQ was used as it examines all components of WHO-defined health literacy, including social and communication components. The HLQ is a 44-item survey that captures the concept of health literacy across nine unique areas (assessed using one scale per domain). Due to the breadth and complexity of this construct, the HLQ generates nine discrete scores rather than a single aggregate health literacy score. NVS was used in two studies [19,24]. The NVS is a validated and commonly used tool to assess health literacy. ARC/LaTrobe and SHS/Missouri instruments include questions regarding fundamental reproductive biology as well as STI pathogens and their symptoms, which were used by the same author in two studies [22,23]. The SHS/Missouri tool also assessed literacy on the efficacy of several risk-reduction measures against STIs and unintended pregnancy [22].

Regarding self-developed questionnaires, in one study in Thailand, the Ministry of Public Health developed a health literacy scale for the prevention of unwanted pregnancies among Thai female adolescents [26]. The questionnaire consisted of 79 questions separated into four sections, with the health literacy for unintended pregnancy prevention section including 35 questions. A study by Lee and colleagues [21] developed a 5-item questionnaire adapted from the National Cancer Institute’s vaccination guidelines to assess participants’ awareness of current HPV vaccination literacy. Another study used a tool based on a previous study in which scale items were built to directly reflect WHO definitions of communicative and critical health literacy to investigate health literacy regarding the HPV vaccine based on five factors (three communicative health literacy and two critical health literacy items) [25]. One study assessed health literacy using a single item – confidence level when filling out medical forms – which was self-developed and validated by another author [20]. Responses to this question were presented as ‘not at all,’ ‘a little,’ ‘sometimes,’ ‘quite a bit,’ and ‘extremely.’

For the two qualitative studies, one article developed an interview guide based on the Integrated Model of Health Literacy, Social Cognitive Theory, and Diffusion of Innovations theory [27]. The second study used the European Health Literacy Project (HLS-EU) as a guide for focus groups [28].

### Health literacy interventions

To foster better SRH among higher education students, two intervention-related studies (*n* = 2) discussed technology-based interventions on students’ SRHL. The first study [27] was a systematic evaluation of an interactive web-based sexual health literacy intervention called ‘Smart Girlfriend.’ It was conducted using a multicenter randomized controlled trial on 781 unmarried female university students (≥18 years old) at 5 universities with dormitories in Hong Kong. The intervention was designed as a sexual health literacy intervention empowering female university students with enhanced knowledge, attitudes, norms, and self-efficacy around managing sexual health, particularly condom use for safe sex practice.

‘Smart Girlfriend’ was delivered in three phases taking 30 min of engagement with online information. The information flow was based on the Health Belief Model and the Continuum of Conflict and Control theory, beginning with assessing students’ perceived susceptibility and perceived severity for individual STIs through a validated 8-question index. Once students obtained personalized results including factors that may increase or reduce their risk of acquiring STIs and cervical cancer, they were provided with knowledge-based information regarding STIs, cervical cancer, condom use procedures and tips, as well as web links for local STI testing, cervical screening programs, and HPV vaccination programs. In the final phase, participants received a summary of individual factors facilitating decision-making about consistent condom use in future sexual activities and were asked to rate their level of self-efficacy in terms of knowledge, skills, clarity of information, and perceived support and advice on a scale of 1–5.

Participants were asked to self-report consistency of condom use with every partner at 3-month and 6-month follow-up assessments. Results indicated that consistency of condom use increased over time in both groups. The intervention group exhibited 5% higher consistency at the 3-month follow-up and 1% higher consistency at the 6-month follow-up compared to the control group; however, these differences were not statistically significant.

The second study [29] explored health literacy needs and preferences for a technology-based intervention (app) to improve SRH among college students. Researchers used an interview guide to elicit participants’ preferences for an eHealth intervention utilizing user-centered design with potential for greater usefulness and engagement. The proposed app would be used before, during, and after campus health clinic visits, aiming to help students access credible and patient-centered information, understand SRH information, know what questions to ask their provider, and facilitate making decisions about their SRH aligned with their needs and health goals.

The research used an interview guide based on the Integrated Model of Health Literacy (IMHL), Diffusion of Innovations Theory (DOI), and Social Cognitive Theory (SCT). This qualitative study employed semi-structured, in-depth interviews with 20 college students. Dominant themes related to accessing health information and services, evaluating options to make decisions, intervention utility and characteristics, and the emergent theme of credibility.

Participants suggested various methods for information delivery, including facts, statistics, infographics, pictures and images, videos, and interactive games or quizzes. Furthermore, participants expressed the need to include information on health services available to students – on-campus, off-campus, and those accessible regardless of insurance status. Regarding access to SRH services, most participant responses focused on STI testing. Participants commonly discussed the app’s role in helping with contraception decisions. They also expressed interest in engaging communication platforms within the app to interact with healthcare providers and peers, with several suggesting open forums or discussion boards. Participants reported the app could educate partners together, remind them to schedule appointments, and encourage contraceptive use or STI testing.

## Discussion

This scoping review aimed to map available literature related to SRHL among higher education students and identify gaps for future research and practice. The review explored health literacy as both an outcome and exposure regarding SRH, identified measurement tools to assess students’ SRHL, and examined interventions implemented in university settings.

Our findings showed that SRHL was adequate in the reviewed studies, whereas a previous systematic review on the same topic in sub-Saharan Africa concluded that there is a lack of SRHL among young people [30]. This difference could be due to several factors including cultural and religious values related to sexual and reproductive health, sex education, and sexual practices of participants from different regions. Moreover, the majority of studies are from the United States, with a considerable lack of research from other regions such as South America, Africa, and the Middle East and North Africa. Only two qualitative studies from the United States are included in this review, highlighting the need for more contextualized research that will help explore sociocultural, religious, and environmental factors influencing SRHL in different settings.

As an outcome, SRHL was determined by factors embedded at different levels including individual, behavioral, and interpersonal levels. Individual factors include female sex, older age, certain regions, sexual orientation, religious affiliation, school year, field of study, and age of first sexual experience. Females had significantly greater health literacy compared to males, showing consistency with another study conducted among adolescent refugees which revealed that females had more health literacy on STIs compared to males [31]. This could be due to increased focus on female sexual health interventions for enhancing knowledge and awareness on SRH [32] due to biological differences, as women are more susceptible than males to certain SRH conditions [33].

Students from East and South Asia exhibited lower SRHL. Cultural norms in these regions tend to discourage open discussions about sexual health, implying that university students have limited access to sexual health education. For example, in China, traditional values emphasizing sexual modesty can impede comprehensive sex education, leaving gaps in students’ knowledge about sexual health [34]. Similarly, in Nepal [35], societal taboos surrounding sexuality make it difficult to implement effective sexual health education programs, leaving university students with insufficient knowledge. These cultural barriers impede students’ access to accurate information, increasing their vulnerability to sexual health risks.

Health literacy was also found to be lower among certain religious groups. This could be explained by conservative views on sexuality existing throughout some religions, which serve as deterrents against early onset of sexual experiences or delay sexual activity until marriage [36,37]. However, we should be cautious about interpreting findings related to religion’s role in influencing SRHL, especially when considering questions applied in measurement tools regarding unintended/unplanned pregnancies, STIs, condom use for safe sex, etc. In some religions such as Islam and others, premarital sex is prohibited, and Western sexual health values contradict Muslim cultural values. Thus, Muslim participants might avoid answering these questions since they do not align with their Islamic sexual values [38,39]. Consequently, we cannot consider their SRHL low compared to participants with other religions.

These findings highlight the importance of considering religion and culture as crucial factors when planning interventions to enhance SRHL among young people. The MENA region’s contentious approach to sex education is rooted in cultural taboos rather than Islamic theology. To ensure effective application, Western techniques must be tailored to the region’s particular history, religion, and culture. Integrating sex education within these frameworks necessitates a flexible, evidence-based approach that allows for change and cultural adaptability [39].

Regarding behavioral factors, students with sufficient sexual education, those with higher numbers of sexual partners during their lifetime, and those engaged in different sexual practices demonstrated higher sexual health literacy. This could be due to increased personal sexual experience, which enhances an individual’s sexual health literacy.

An interpersonal factor identified in this review involves sexual topics being considered taboo within families. Sexuality is considered taboo in many cultures, which may lead to lack of acceptability for sex education, resulting in limited awareness of sexual-related topics among young people [39]. We also need to be careful about interpreting the role of family in shaping SRHL across regions with diverse religions and cultures. Muslim mothers in Western cultures might be open to discussing Islamic sexual health values with their daughters, yet prohibit them from attending sex education in schools to avoid exposure to Western sexual health values that conflict with Islamic values [40].

Health literacy as an exposure was associated with various outcomes including HPV knowledge and confidence, HPV vaccine knowledge, and sexual attitudes. Results were consistent with findings from a behavioral risk factor surveillance system study where health literacy was positively associated with HPV vaccination completion [41]. It showed that higher levels of health literacy increased uptake of at least one dose of the HPV vaccine. Conversely, another review reported that health literacy did not significantly influence vaccination behavior. However, that review aimed to summarize evidence on the relationship between health literacy and both vaccination intentions and status regardless of vaccine type or age group [42]. Our review also showed that higher levels of health literacy help prevent unwanted pregnancy. Another study found consistent results [26], indicating that college students with low health literacy were more likely to exhibit inappropriate behaviors concerning unintended pregnancy prevention.

Since health literacy is shaped by context, different assessment tools may be needed in different settings. Consequently, studies included in this review used various assessment tools. Eight studies used different SRHL measurement tools that were validated considering contextual needs. A total of nine instruments were used to evaluate SRHL quantitatively, compared to only two qualitative research studies.

The rapidly evolving health information landscape has led to increased concerns regarding young people’s relationships and sexual practices, particularly regarding the internet’s impact on their safety and sexual behaviors [43]. The ease with which young people can access sexually explicit content online and on social media is another concern, as it facilitates practices like sexting that may impact attitudes, behaviors, and sexual wellbeing [44]. Therefore, evidence-based sexual health literacy interventions are essential for ensuring adolescents receive correct and accurate health information. In our review, only two eligible studies included assessment of technology-based interventions on students’ SRHL literacy to promote improved SRH among college students.

These studies focused on enhancing knowledge, attitudes, norms, and self-efficacy around managing sexual health, particularly condom use for safe sex practice. The results showed positive effects on outcomes but were not statistically significant. These findings are consistent with existing studies related to digital-based interventions. A 2022 review among adolescents and young adults [45] concluded that online social media (Facebook) and other technology-based strategies (computer, texting, web-based, internet-based) have shown positive but not statistically significant effects, particularly in modifying misconceptions and negative perceptions and promoting safe sexual practices (condom usage, abstinence, STI screening/follow-up). In the same review, researchers found that improving knowledge and practice results of SRH typically requires more than just texting. Text messaging in conjunction with other tactics – such as incorporating behavioral change techniques or curricula, offering financial incentives, narrating educational stories, using video messaging, providing psychosocial support or counseling, using Q&A bidirectional texting, and using screening – is more likely to result in higher screening, follow-up, and positive SRH outcomes.

### Conceptual model for SRHL

**Figure 2.**
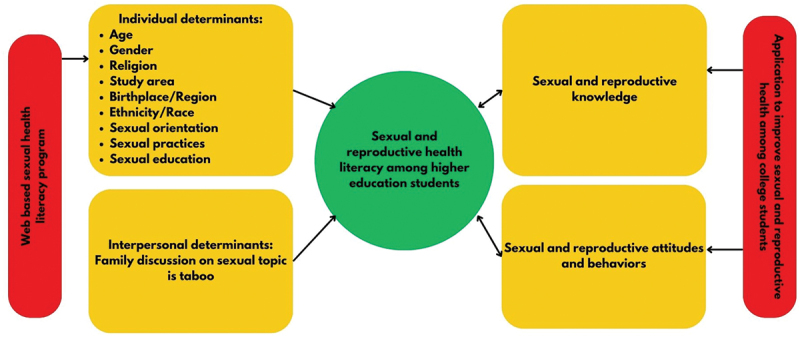
Conceptual model of reproductive and sexual health literacy.

To enhance the visual representation of findings from this review, we constructed a conceptual model (Figure 2). Our findings showed that SRHL among higher education students was researched both as an outcome and as an exposure. SRHL as an outcome was positively influenced by non-modifiable factors including age over 20 years, female sex, and other individual factors such as field of study, sexual orientation, sexual practices, and sexual education. Participants who identified as Catholic reported higher SRHL compared to those unaffiliated with religion, unaffiliated but spiritual, Protestant and other non-Catholic Christians, Buddhists, Muslims, or Hindus. Moreover, specific regions, races, and ethnicities were among factors that negatively affected SRHL. On the interpersonal level, as with other health behaviors and lifestyle factors for this generation [46,47], family should play a crucial role in shaping SRHL. However, our review results showed that family discussions related to sexual health were considered taboo, negatively influencing SRHL levels among young people.

Our model also reflected on the need to plan personalized and contextualized interventions as reported in the literature. One intervention involves an application that would assess students’ SRH needs and tailor assessment findings toward effective health education, promotion interventions, and resources. This intervention would enhance sexual and reproductive knowledge, attitudes, and behaviors, thus improving students’ SRHL. The second intervention is a web-based SRHL program designed to promote sexual education and practices among students.

### Strengths and limitations

This review is the first to explore SRHL among higher education students. A main strength of our review is its focus on studies that explicitly examine the concept of SRHL among this generation, which exposed us to specific literature, validated tools, and interventions that support future research and practice in this area. The comprehensive initial search we conducted about SRHL among this population ensured we included the most updated literature on this topic.

However, our review has some limitations, including that we only included English, primary research studies that explicitly mention the concept of SRHL. Excluding articles in other languages, different research designs, and gray literature may introduce biases and prevent us from exploring other contexts and cultures that would enrich our understanding of the topic across diverse populations of the young generation. The majority (eight studies) of the included studies employed a cross-sectional approach, which prevents us from establishing causal relationships. Finally, given that most studies focus on females, we recommend more research on SRHL in males, as there are few studies on this population.

### Implications for research, policy, and practice

Our review identifies critical gaps in the current literature and offers strategic directions for future research, policy, and practice. Given that SRHL is a multifactorial determinant of health for the younger generation, a concerted focus on comprehensive research is essential. More rigorous quantitative and qualitative studies are urgently needed to establish SRHL as a key determinant of reproductive health outcomes among college students. The current evidence base particularly lacks studies exploring contextual and environmental factors – such as cultural norms, social influences, religious values, and institutional contexts – that shape SRHL in higher education settings. More research is needed among male students in higher education, as the majority of studies have been conducted among female students.

To address these gaps, contextual and qualitative research should be prioritized to better understand how sociocultural dynamics influence SRHL for this demographic. Additionally, exploring the role of family in reproductive health communication, its impact on youth SRHL, and the barriers that hinder such communication are crucial areas for further investigation. Such insights can guide the design of family-centered interventions that enhance intergenerational dialogue on SRH issues.

From a policy perspective, there is an urgent need to develop evidence-based, culturally tailored interventions that leverage behavioral and cognitive theories to improve SRH knowledge and practices. Academic institutions should adopt validated assessment tools for measuring SRHL among students, enabling targeted health education and promotion programs. Moreover, our findings underscore the potential of technology-driven interventions, which can effectively assess student needs and enhance SRHL on campuses.

Considering these insights, we recommend integrating SRHL into educational curricula and developing policy frameworks that support family-based interventions, enabling more effective reproductive health communication between young people and their families. Policymakers, educators, and health professionals must collaborate to ensure that the next generation is equipped with the knowledge, skills, and resources necessary to make informed SRH decisions in an increasingly complex social landscape.

## Conclusion

This scoping review maps available research related to SRHL among higher education students and highlights some validated tools and effective, technology-based interventions. Contextual research exploring the sociocultural, religious, and environmental factors influencing SRHL of the young generation is needed to provide evidence for interventions. Family-based health communication interventions are fundamental to enhancing SRHL in this population.

## List of abbreviations

SRH: Sexual and reproductive health
SRHL: Sexual and reproductive health literacy
HL: Health literacy
REALM: Rapid Estimate of Adult Literacy in Medicine
TOFHLA: Test of Functional Health Literacy in Adults
PRISMA-ScR: Preferred Reporting Items for Systematic Reviews and Meta-Analyses extension for Scoping Reviews
STIs: Sexually transmitted infections
HLQ: Health Literacy Questionnaire
NVS: Newest Vital Sign
ARCSHS: Australian Research Centre In Sex, Health, and Society
SHS: Sexual Health Survey
HLS-EU: European Health Literacy Project
IMHL: Integrated Model of Health Literacy
DOI: Diffusion of Innovations Theory
SCT: Social Cognitive Theory
KAP: Knowledge, attitudes and behaviors/practices
